# A Humeral Osteosarcoma Mimicking Osseous Leiomyosarcoma: A Case Report

**DOI:** 10.7759/cureus.52469

**Published:** 2024-01-17

**Authors:** Yi Yan, Victoria Xie, David Perrin, Miao Lu, Laurence Stillwater

**Affiliations:** 1 Medical Imaging, St. Joseph's Health Care London, London, CAN; 2 Diagnostic Radiology, University of Manitoba, St. Boniface General Hospital, Winnipeg, CAN; 3 Rady Faculty of Health Sciences, University of Manitoba, Winnipeg, CAN; 4 Surgery, University of Manitoba, Winnipeg, CAN; 5 Pathology, University of Manitoba, Winnipeg, CAN

**Keywords:** pathological fracture, osteoid matrix, conventional osteosarcoma, leiomyosarcoma, ct guided biopsy

## Abstract

Osteosarcoma stands as one of the primary mesenchymal bone neoplasms commonly encountered in clinical practice. This malignancy often presents with a wide range of distinctive imaging characteristics. Here, we present a unique case wherein a delayed diagnosis of high-grade osteosarcoma occurred due to the absence of an osteoid matrix in the initial imaging studies.

A 61-year-old female, initially presented with a left humeral fracture. As the healing of the fractured bone was delayed and the possibility of a pathologic fracture was considered, a CT-guided biopsy was performed. Histological examination of the biopsy sample initially suggested an osseous leiomyosarcoma. The lack of osteoid matrix on radiographs including aggressive intra-medullary mass seen on MRI, combined with the patient’s age, appeared consistent with a diagnosis of leiomyosarcoma of bone. As a result, the initial diagnosis was not called into question. Due to neurovascular involvement, this led to a forequarter amputation. However, upon microscopic examination of the amputation specimen, certain areas exhibited features indicative of malignant osteoid deposition, ultimately supporting a revised diagnosis of high-grade osteosarcoma.

This case underscores the critical importance of considering the limitations of core biopsy samples, especially when dealing with suspected limb masses associated with pathological fractures. Radiographs and CT scans can prove invaluable in ruling out subtle adjacent osteoid, and ultimately a multidisciplinary approach to the diagnosis of osteosarcoma is imperative to ensure accurate identification.

## Introduction

Osteosarcoma, also referred to as osteogenic sarcoma, stands as the predominant form of bone cancer originating within bone tissues, with a worldwide annual incidence rate of 3.4 cases per 100,000 individuals [1,2]. This malignancy accounts for as much as 20% of all primary bone cancers [3]. It tends to manifest more frequently in individuals within* the age range of 10 to 30 years,* primarily affecting children, adolescents, and young adults, thereby ranking as the third most prevalent cancer in the adolescent population, trailing only behind lymphomas and brain tumors [1,2,4]. Osteosarcoma commonly develops in close proximity to the growth plates in the long bones of the appendicular skeleton, such as the femur. It typically presents with progressive pain, often linked to the rapid expansion of the tumor and local bone destruction. Additionally, secondary osteosarcomas may arise within areas previously exposed to radiation or as a result of secondary degeneration in cases of Paget's disease and fibrous dysplasia.

The primary diagnostic tool for evaluating primary bone tumors is typically plain radiography [5]. Osteosarcoma lesions can present as entirely osteolytic (in approximately 30% of cases), osteoblastic (in about 45% of cases), or a combination of both [3,4]. An elevation of the periosteum may manifest as a Codman triangle or a sunburst appearance. When it comes to assessing the primary lesion, magnetic resonance imaging (MRI) proves to be the most effective method, providing comprehensive insights into intramedullary disease, soft tissue extension, and the presence of local skip lesions. Furthermore, MRI excels in characterizing the architectural features of the tumor [4]. For evaluating the response to chemotherapy, fluorodeoxyglucose positron emission tomography (FDG PET) can be employed, while technetium-99m methylene diphosphonate (Tc-99m MDP) bone scanning is valuable for detecting distant bone metastases [4].

Microscopically, osteosarcoma is characterized by the formation of an osteoid matrix [6]. The World Health Organization classifies osteosarcoma of the bone into eight categories, each distinguished by unique biological behaviors and clinical outcomes: conventional, telangiectatic, small cell, low-grade central, secondary, parosteal, periosteal, and high-grade surface. The most prevalent subtype is conventional osteosarcoma, accounting for 70-80% of cases [7]. Typical treatment involves initiating chemotherapy before surgery, followed by surgical removal of the tumor, and subsequently, further chemotherapy [7]. Surgical procedures often aim to preserve the affected limb, but in some cases, amputation may be necessary, guided by imaging and staging assessments. The presence of metastatic disease at the time of diagnosis and the response to chemotherapy stands as the most critical factors influencing the prognosis of the disease [7]. Osteosarcoma typically exhibits resistance to radiation, with radiation therapy usually reserved for unresectable disease or palliative care [7]. Achieving gene target therapy for osteosarcoma has proven to be challenging thus far due to the frequent occurrence of gene amplifications and significant genetic heterogeneity [8].

High-grade conventional osteosarcoma is recognized for its aggressive nature. In this report, we present an exceptional case where high-grade conventional osteosarcoma was initially misdiagnosed as leiomyosarcoma due to the absence of osteoid matrix in the initial imaging studies and the constraints of the biopsy sample.

## Case presentation

**Figure 1:**
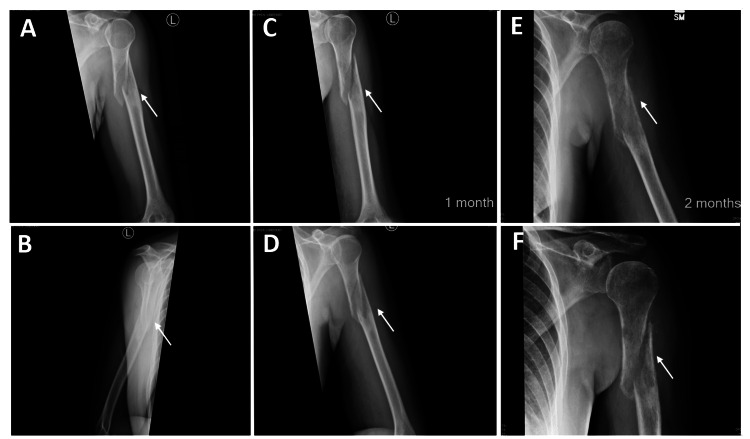
Series of radiographs explicit the progression of pathological fracture at the proximal third of the humeral shaft A, B: Initially frontal and later radiographs showed an acute oblique fracture of the proximal diaphysis of the left humerus with approximate half shaft width and lateral displacement of the distal fracture fragment. C, D: One-month follow-up radiograph again showed a mild interval improvement of alignment of the fragments. The distal component remains ununited and displaced laterally. E, F: Two-month follow-up radiograph showed unaltered alignment and positioning with ongoing mild impaction and apex medial/anterior angulation. No Significant interval callous formation. The union is not complete. There appears to be a moth-eaten pattern of the adjacent bony matrix concerning pathological fracture.

A 61-year-old woman in good health sought care at the urgent care center for a minor injury to her left arm. A radiographic examination revealed a mildly displaced spiral fracture in the upper third of the left humerus (Figures 1A, 1B). She was subsequently referred to the orthopedic clinic for further evaluation. Follow-up radiographs conducted at one- and two-month intervals post-injury raised concerns of a potential pathologic fracture. These images depicted a non-healing fracture and an increasingly irregular appearance suggestive of an underlying bone abnormality (Figures 1C-1F). Consequently, the patient was referred to orthopedic oncology, where a core needle biopsy guided by imaging was recommended. At this stage, both primary and metastatic lesions were under consideration. Further investigations included metastatic workup and additional characterization imaging studies.

**Figure 2:**
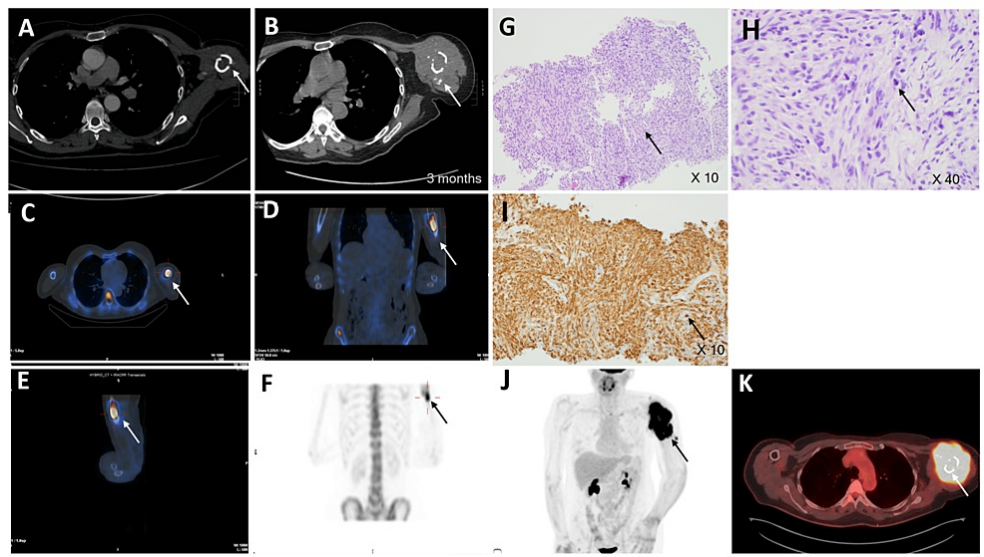
Left humeral aggressive bony lesion by CT, bone scan, and PET A, B: Two separate CTs from the chest (part of metastatic workup) and during the biopsy, respectively, showed a non-united fracture with interval development of extraosseous/heterotopic ossification in two months. C-F: A whole-body skeletal survey was performed. Intense heterogeneous activity to the fracture involving proximal left humerus with questionable increased marrow density. The possible pathological fracture is again raised. G: The biopsy H/E consisted of a limited amount of material. The histological analysis of the biopsy sample revealed a spindle cell neoplasm characterized by moderate cytologic atypia (arrow), (10x). H: Biopsy H/E sample revealed a spindle cell neoplasm showed scattered mitotic figures (arrow), (inset 40x). I: Caldesmon immunohistochemical stain demonstrated strong diffuse positivity (10x). J, K: A near whole body scan (base of the skull to mid-thighs) FDG scan has been obtained supplemented with an uninfused low-dose CT for attenuation correction and anatomic localization. Intense activity to the large soft tissue mass surrounding the pathological fracture involving the proximal half of the left humerus as described consistent with a malignant process. It was in keeping with biopsy-approved osseous leiomyosarcoma. FDG: Fluorodeoxyglucose

Computed tomography (CT) scans of the chest, abdomen, and pelvis were performed, revealing no evidence of other primary or metastatic disease. The initial CT chest examination confirmed a non-united humeral fracture with a suspected underlying lesion (Figures 2A, 2B). A whole-body bone scan was also conducted, showing intense and irregular activity at the site of the fracture, along with potential increased marrow density (Figures 2C-2F). This prompted renewed consideration of a pathological fracture. Given the solitary nature of the lesion and the revised impression that metastatic disease was no longer likely, alternative diagnoses were considered. Subsequently, a CT-guided biopsy was performed on the left humerus. At this juncture, the CT scan indicated a non-united fracture with suspected extraosseous ossification that had developed since the initial CT examination (Figures 2A, 2B).Under CT guidance and using coaxial technique, a 19 gauge introducer was placed into the soft tissue part of the lesion and 5 x 20 gauge core biopsies were obtained. The biopsy consisted of a limited amount of material, including several tiny cores of tissue measuring 0.4 x 0.4 x 0.1 cm in aggregate. The histological analysis of the biopsy sample revealed a spindle cell neoplasm characterized by moderate cytologic atypia (Figure 2G) and scattered mitotic figures (Figure 2H). Notably, osteoid tissue was not identified in the examined material. The tumor cells exhibited widespread immunoreactivity for smooth muscle actin (SMA) and caldesmon (Figure 2I), while another muscle marker, desmin, tested negative. These combined histological and immunohistochemical findings conclusively diagnosed the condition as leiomyosarcoma. Because it is unusual to have primary bone leiomyosarcoma, we thought that it might be metastatic from gynecologic origin, so a positron emission tomography (PET) scan was ordered. The PET scan demonstrated the absence of a primary pelvic mass but revealed high metabolic activity associated with a large soft tissue mass surrounding the pathologic fracture of the left humerus (Figures 2G-2H). In summary, both the CT scans of the chest/abdomen/pelvis and the PET scan failed to provide evidence of a primary site of disease*.*

**Figure 3:**
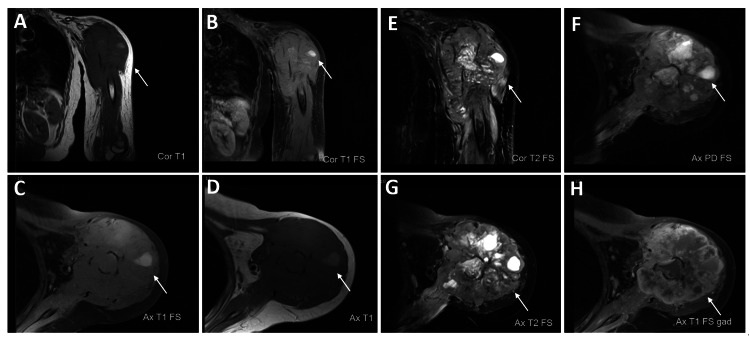
Further characterization of left humeral aggressive bony lesion A-H: The known mass lesion about the proximal humerus is again demonstrated. It extends to involve the humeral head and extends inferiorly to the level of the mid-humeral shaft. This measures approximately 10.3 cm anterior-posterior by 8.3 cm medial-lateral by 15.9 cm craniocaudad. This replaces the marrow about the proximal humerus and there is associated soft tissue extension and pathologic fracturing. The lesion is predominately hypointense/isointense on T1-weighted sequences with some T1 hyperintense signal of the lateral deltoid region (A-D). The lesion is heterogeneous on fluid-sensitive sequences and there is an avid heterogeneous enhancement (E-H). This is in keeping with the known leiomyosarcoma.

To gain a more comprehensive understanding of this solitary humeral lesion, an MRI was subsequently ordered. The MRI reaffirmed the presence of a mass lesion in the proximal humerus, which had replaced the marrow and extended into the surrounding soft tissues, causing pathologic fracturing. The lesion predominantly exhibited hypointense to isointense signals on T1-weighted sequences, along with some T1 hyperintense signals in the lateral deltoid region, suggesting the presence of internal hemorrhages (Figures 3A-3D). On fluid-sensitive sequences, the lesion displayed heterogeneity, and there was pronounced and varied enhancement (Figures 3E-3H). These findings, while non-specific, were consistent with characteristics typically seen in sarcomatous lesions.

Subsequently, the case underwent a thorough multidisciplinary review by the local sarcoma tumor board. The medical oncology team determined that neoadjuvant systemic treatment was not warranted. The orthopedic oncology team presented surgical treatment options, which normally would perform upper limb disarticulation. However significant neurovascular involvement led to a forequarter amputation with an extremely challenging limb salvage procedure. The limb salvage procedure was associated with a high risk of morbidity, the potential for positive margins, post-operative complications, and a significant impairment of upper extremity and hand function. After comprehensive discussions regarding these options, the patient decided to undergo an amputation. Consequently, the patient underwent a successful wide resection, resulting in a left forequarter amputation to address the leiomyosarcoma originating from the humerus.

**Figure 4:**
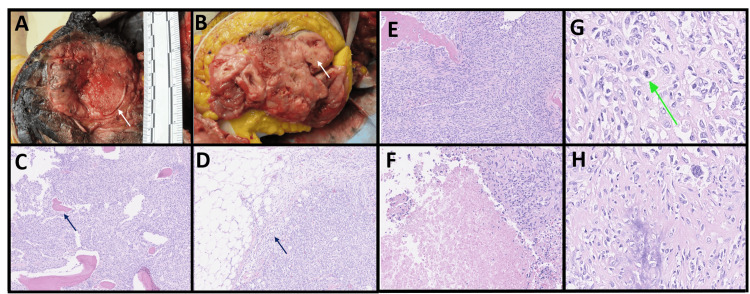
Gross and microscopic findings of perinephric tumor A, B: Gross images showed a tumor arising from the shaft and head of the humerus (white arrow) demonstrating a white-tan, circumscribed soft, hemorrhagic, partly necrotic tumor, with smooth lobulated borders (white arrow). Areas of necrosis account for approximately 30% of the tumor. The mass extends through the articular surface of the humerus. C: 10x low power showing tumor infiltrating the existing bone as indicated in arrow. D: 10x low power showing tumor involving the soft tissue as indicated in arrow. E: 20x spindled cells in fascicular pattern as indicated in arrow. F: 20x tumor necrosis as indicated in the arrow. G: 40x mitotic figure as indicated in arrow. H: 40x tumor in the soft tissue with osteoid matrix as indicated in arrow.

The surgical specimen was subsequently sent to the pathology department for evaluation. Grossly, the tumor arose from the shaft and head of the humerus, displaying a well-defined soft, hemorrhagic, partly necrotic appearance with smooth, lobulated borders (Figures 4A, 4B). It measured 18.5 cm x 10.0 cm x 12.5 cm (SI x CC x ML), with approximately 30% of the tumor showing areas of necrosis. The tumor extended through the articular surface of the humerus and came into close contact with it but did not involve the scapula. Under microscopic examination, the tumor was found to infiltrate the existing bone and involve the surrounding soft tissue (Figures 4C, 4D). Broad sections of the tumor exhibited characteristics akin to those seen in the initial biopsy tissue, featuring spindle cells with moderate cytological atypia and scattered mitoses (Figures 4E-4G). Immunohistochemical analysis revealed a non-specific myofibroblastic pattern of SMA positivity, with negative results for desmin and caldesmon. However, there were also areas within the tumor displaying the presence of osteoid matrix, a diagnostic hallmark of osteosarcoma (Figure 4H). Consequently, the diagnosis was revised to osteosarcoma. This re-evaluation prompted further discussion at the multidisciplinary tumor board, leading to the initiation of three cycles of adjuvant chemotherapy including cis-platinum/doxorubicin as the next course of action. She had a one-year follow-up showing no recurrent disease thus far.

## Discussion

We present a case involving a 61-year-old female diagnosed with conventional osteosarcoma within the context of a non-healing displaced humeral fracture. Initially, an osseous leiomyosarcoma diagnosis was established based on a CT-guided biopsy specimen, primarily due to the presence of smooth muscle-like spindle cell proliferation, immunohistochemical panel results, and the absence of osteoid. Given the patient's age and X-ray findings, which aligned with leiomyosarcoma characteristics, there was no initial reason to question this diagnosis. However, the diagnosis was subsequently revised to high-grade conventional osteosarcoma following the identification of malignant osteoid in the surgical specimen, in conjunction with the progressive radiographic evidence of osteoid formation. The spindle cell component of osteosarcoma can indeed exhibit overlapping features with leiomyosarcoma. Thus, the pivotal factor in distinguishing between these two entities is the identification of osteoid, which may require extensive sampling of either the biopsy specimen or radiological findings. Our case notably underscores that sarcomatous tumor imaging findings may overlap due to potential delays in osteoid formation. Given the patient's age and the compatibility of X-ray, CT, and MRI findings with the initial leiomyosarcoma diagnosis, there was no initial questioning of the biopsy diagnosis. There were no detrimental effects on the patients due to the initial misdiagnosis. The patient would have undergone neoadjuvant chemotherapy if this was an osteosarcoma from the start. She however received post-surgery chemotherapy which, so far, has not shown any detriments in terms of local recurrence or metastatic disease. This report highlights the limitations inherent in standard imaging-guided biopsies of bone lesions and emphasizes the necessity of a multidisciplinary approach for achieving an accurate osteosarcoma diagnosis.

Osteosarcoma frequently occurs in children and young adults. Some of the literature suggests that osteosarcoma has a bimodal distribution at the ages of 10-14 years and older than age 60 years [8,9]. The first peak of osteosarcoma is thought to be attributed to increased bony proliferation during puberty [9]. Certain authors suggest the second peak incidence is likely associated with Paget’s disease of the bone and probably represents a distinct biological process [8]. In our very unusual case, it occurred in a female older than 60, with no underlying primary bone disease identified. The exact etiology of this case is not known.

Differentiating osteosarcoma from other types of sarcomatous tumors through imaging can indeed be quite challenging. Osteosarcoma presents a wide range of imaging characteristics, and radiography is often the primary tool for assessing local disease and primary bone tumors. In this particular case, the initial radiograph did not reveal aggressive periosteal reactions like the Codman triangle or sunburst appearances. It was only on subsequent follow-up radiographs that a moth-eaten matrix pattern was suspected, albeit in hindsight (Figures 1B, 1C). Nevertheless, aside from imaging findings, the non-healing aspect of the condition raised a red flag for a possible pathological fracture. Additionally, further inquiry by the clinician revealed that the initial injury was quite minor, occurring while the patient was carrying a gardening box. While MRI is known for its superior ability to characterize tumor structure, such as identifying cystic spaces in telangiectatic osteosarcoma and the presence of multifocal disease, it did not yield any additional specific information in this case and presented rather nonspecific findings for a sarcomatous lesion.

One of the valuable modalities for diagnosing osteosarcoma is dedicated CT scanning, although it was not performed in our case (only metastatic CT chest and CT biopsy were conducted). A meticulous examination for the presence of osteoid matrix or ossification at the fracture site is crucial, as they can offer additional insights beyond what MRI provides. In this instance, a follow-up CT scan revealed subtle, amorphous, densely calcified fragments along the proximal shaft of the humerus, adjacent to the fracture site, in hindsight (Figure 2B). These fragments were not evident in prior images at the time of the fracture. The intramedullary involvement observed was most suggestive of osteoid formation, rather than fractured fragments. The appearance did not resemble the snowflake-like pattern typically seen in chondrosarcoma, making chondrosarcoma much less likely. Furthermore, there was a noticeable and rapidly expanding soft tissue component with necrosis within the mass, further raising the possibility of a high-grade tumor.

Moreover, in this case, the biopsy sample unfortunately failed to provide a comprehensive representation and led to the misdiagnosis as a bone leiomyosarcoma for several reasons. Primarily, the core biopsy specimens were exceedingly limited in scope, rendering them insufficient to encompass the entire lesion, which likely consisted of osteolytic, osteoblastic, and soft tissue components. In hindsight, we only obtained samples from the peripheral soft tissue component of the tumor, overlooking the central osteoblastic part. This discrepancy arose as the radiologist was unable to distinguish fractured fragments from osteoid formation at the fracture site. To potentially resolve this diagnostic dilemma, multiple biopsy passes should ideally be taken from both the lytic and sclerotic regions, as well as the most aggressive soft tissue component, to ensure a more accurate representation of the lesion [10]. The inherent heterogeneity of the tumor certainly amplifies the complexity of making an accurate diagnosis when dealing with limited and small biopsy samples. Furthermore, the initial imaging of the intra-medullary lesion, coupled with the patient's age of over 60, strongly aligned with the characteristics typically associated with bone leiomyosarcoma. Consequently, there was no immediate reason to question the initial diagnosis. It's worth noting that a bone leiomyosarcoma can genuinely mimic the presentation of osteosarcoma. In our case, two muscle markers, specifically SMA and caldesmon, yielded positive results in the biopsy specimen, while only SMA exhibited positivity in the resection specimen. On the other hand, a third muscle marker, desmin, consistently tested negative in both specimens. In retrospect, the SMA positivity exhibited a myofibroblastic pattern, which was considered nonspecific. In the absence of malignant osteoid in the biopsy sample, the tumor did not meet the criteria required for an osteosarcoma diagnosis.

In summary, our case is unique in the literature as an osteosarcoma mimicking a bone leiomyosarcoma on initial imaging and biopsy samples. The absence of representative osteoid was a significant contributing factor to this diagnostic challenge. Interestingly, there have been published cases where high-grade leiomyosarcomas have exhibited a remarkable transformation, with a nearly complete transition into osteosarcomas by identifying areas demonstrating osteosarcomatous dedifferentiation within soft tissue sarcomas [11,12]. The origin of such heterotopic ossifications would be key for the diagnosis of osteosarcoma versus high-grade leiomyosarcoma with osteosarcomatous dedifferentiation and prognoses for these two entities are drastically different. Once more, our case underscores the vital importance of a multidisciplinary approach, particularly in referral centers equipped to offer comprehensive care. In such settings, collaboration among orthopedic surgeons, medical oncologists, histopathologists, radiologists, and radiation oncologists facilitates both reaching an accurate diagnosis and executing definitive treatment plans. It's worth noting that clinical and radiological presentations often offer crucial insights for distinguishing osteosarcoma from other conditions, especially in cases where biopsy samples may not fully represent the underlying pathology.

## Conclusions

This case acknowledges the constraints associated with core biopsy samples, particularly when addressing limb masses accompanied by pathological fractures. Radiographs and CT scans have emerged as invaluable tools for eliminating the possibility of osteoid. In the end, a multidisciplinary approach to osteosarcoma diagnosis becomes imperative to secure a precise and reliable identification.
